# Nanoscale Science and Technology and People with Disabilities in Asia: An Ability Expectation Analysis

**DOI:** 10.1007/s11569-012-0148-0

**Published:** 2012-08-01

**Authors:** Gregor Wolbring, Natalie Ball

**Affiliations:** Department of Community Health Sciences, Specialization Community Rehabilitation and Disability Studies, Faculty of Medicine, University of Calgary, 3330 Hospital Drive NW, Calgary, AB T2N4N1 Canada

**Keywords:** Ableism, Ability expectations, Disabled people, Nanoethics, Nanotechnology governance, People with disabilities, Science and technology

## Abstract

Science and technology, including nanoscale science and technology, influences and is influenced by various discourses and areas of action. Ableism is one concept and ability expectation is one dynamic that impacts the direction, vision, and application of nanoscale science and technology and vice versa. At the same time, policy documents that involve or relate to disabled people exhibit ability expectations of disabled people. The authors present ability expectations exhibited within two science and technology direction documents from Asia, as well as in two policy documents generated and influenced by disabled people from Asia. As well, the authors discuss the impact of the ability expectations exhibited in these four documents with respect to the relationship between science and technology and disabled people.

## Introduction

Nanotechnology has three different meanings. As a term, nanotechnology was originally coined to describe a way to manufacture something from atomic molecules (such as the food replicator in many science fiction films where one says, for example, “Coffee” and the machine builds, synthesizes the coffee molecule by molecule) [16]. The terms “Molecular manufacturing" and "molecular nanotechnology" cover this meaning today [31,33]. In 2000, nanotechnology became linked to nanobots and nanoreplicators [11]. Since then, nanotechnology underwent another transformation to mean ‘nanoscale technology’ and ‘nanoscale sciences,’ covering research and development products, ideas and processes with controlled size below 300 nm (some say as small as 100 nm) [31,33]. This latter definition includes the first two meanings and expands it to any nanoscale application products and processes. The use of this meaning for nanoscale was, among others, pushed by the 2001 workshop [12] “Nanotechnology, Biotechnology, Information technology and Cognitive science (NBIC): Converging Technologies for Improving Human Performance,” organized by the National Science Foundation and the Department of Commerce (both USA) which focused on nanoscale. It stated: “The integration and synergy of the four technologies (nano-bio-info-cogno) originate from the nanoscale, where the building blocks of matter are established” [12]. The business plan of the International Organization for Standardization Technical committee 229 (ISO/TC229) on Nanotechnology that is charged to produce standards for classification, terminology and nomenclature, basic metrology, calibration and certification, and environmental issues related to nanotechnology includes the following:

1. Understanding and control of matter and processes at the nanoscale, typically, but not exclusively, below 100 nanometres in one or more dimensions where the onset of size-dependent phenomena usually enables novel applications, where one nanometre is one thousand millionth of a metre,
2. Utilizing the properties of nanoscale materials that differ from the properties of individual atoms, molecules, and bulk matter, to create improved materials, devices, and systems that exploit these new properties International Organization for Standardization (ISO) [9].

The definition used by ISO/TC229 International Organization for Standardization (ISO) [9] covers: a) many different nanoscale science and technology products and processes; b) any science and technology fields with nanoscale aspects; and c) nanoscale-enabled science and technology fields, product and processes*.*

If nano would have stayed with its original meaning, not many would be investing into nanotechnology yet, as the ‘food replicator’ is a long term goal. However, moving the meaning to nanoscale as in the 2001 workshop makes nearly every science and technology field part of the nano story, with many possible products and impacts. Therefore, it is understandable that nanoscale science and technology and its convergence with other technologies generates a lot of interest in many countries, including Asia. Many Asian countries such as India[32], China [10] and others [13] are increasingly involved in nanotechnology. A Global R&D Report, ‘Changes in the R&D Community’ published by R&D Magazine [3] in 2005 puts China in 4th place behind India, Japan and the US in R&D spending [6]. According to a Chinese national nanotechnology coordination committee that was co-established in 2001 by the Ministry of Science and Technology, the CAS and the National Natural Science Foundation China's spending on developing nanotechnology over the past 5 years was more than three times that between 2001 and 2005 China [5].

With spending money, concrete expectations are forthcoming. In the 2010 Malaysian statement on Nanotechnology one reads:” Despite being measured in the billionth of a metre, this revolutionary technology holds immense potential to emerge as a growth engine of the Malaysian economy” [7]. In 2005 at that time, President A.P.J. Abdul Kalam from India outlined his vision for nanotechnology in India as follows [19]:

“The last two centuries have seen rapid development of the chemical age. The advancements made in material science and technology gave the impetus for both the nuclear and biological age to flourish. Succession of these technology periods has involved progression from simpler materials to more complex forms of science and engineering. We are today at the convergence of nano, bio and information technologies. This age, I feel will create a historic revolution and we must be in the driver’s seat to contribute towards this societal change.” [19]

He concluded the column by saying:

“I am convinced that nano is the greatest building block for healthcare, structural material, in electronics, automation, etc, and will become the platform for new cutting edge technologies to grow for the better living of mankind.” [19]

A 2005 survey which included people from India [21] concluded that the top 10 nanotechnology applications for development are:

1. Energy storage, production and conversion;
2. Agricultural productivity enhancement;
3. Water treatment and remediation;
4. Disease diagnosis and screening;
5. Drug delivery systems;
6. Food processing and storage;
7. Air pollution and remediation;
8. Construction;
9. Health monitoring;
10. Vector and pest detection and control.

Due to the broad scope of nano-endeavours, nano-visions impact many aspects of societies and various social groups, disabled people among them, on numerous levels.

However, there are problems with respect to who is shaping the visions and goals of nanoscale science and technologies. According to a 2011 Nature Nanotechnology article, “Public engagement with nanotechnology is not nearly as developed as we once expected it to be by the year 2011” [29], despite the “impressive battery of focus groups, citizen juries, consensus conferences, large-scale surveys and other instruments” [29] that were envisioned to facilitate the Democratization of Nanotechnology and the public engagement. As to the group of disabled people, a group the authors want to highlight in this article, it was noted in 2007 that people with disabilities were not part of these public engagements instruments and not part of the nano-engagement discourse period at that time [34]. In this article, we will update the visibility data related to disabled people in the nanodiscourse using frequency keyword analysis of various databases used in [34] and present data as to the visibility of disabled people in Asia using as data a sample of Asian newspapers namely The Star (Malaysia), China Daily, The Hindu and the Korean Times.

However, this is only one set of data to be presented. Each field enabled by nanoscale science and technologies poses distinct challenges and impacts various segments of society and influences how humans relate to each other on the individual and societal level, locally and globally. At the same time ableism, the favoritism for certain abilities, leads to a particular kind of understanding of one’s self, one’s body and one’s relationship with others of one’s species, other species, and one’s environment [39]. It is not unreasonable to expect that nanoscale science and technology advances enable changes in ability expectations and vice versa. The authors analysed two disability related documents, one nano related document and one science and technology related document through an ableism (favoritism for certain abilities) and ability expectation lens. As to disability related documents, the authors investigated the recent Bali Declaration on Disabled Person adopted at the 19th ASEAN Summit in Dec 2011 [2] and the 2011 Durban declaration from the 8^th^ Disabled People’s International (DPI) World Assembly [1]. Furthermore, the authors analysed the nanotechnology strategy statement from Malaysia [8] as well as a science and technology strategy statement from Thailand Sakarindr [20]. The authors present here what ability expectations are exhibited in these documents and discuss the impact of the ability expectation exhibited in the four documents as to the relationship between science and technology and disabled people.

- Part 1: Visibility

Repeating the frequency analysis of keyword combination of nanotech and various social groups with the databases used in 2007 [34] we found little change between 2007 and 2012 (Table 1).

**Table 1:** Keyword

Keyword	Ovid	Cambridge Scientific Databases including IBSS	Academic Premier Search	Google Scholar (2007/2012)	Google (2007/2012)
Nanotechnology	15137	13125	14592	82,200/538,000	123,000,000/25,500,000
+ Health	1024	566	1862	8,010/71,500	55,500,000/21,400,000
+ Women	133	46	543	2,370/17,300	9860000/8,376,000
+ Disabled people	2	1	9	86/502	31400/686,000
+ People with disabilities	2	0	18	147/485	81800/2,230,000
+ The poor	269	22	691	1,550/14,200	227,000/7,070,000
+ The south	133	6	959	1,020/6,190	614000/11,300,000
+"indigenous people"	0	0	17	125/404	21200/2,380,000
+ Patient	564	75	717	2,480/22,400	2,210,000/3,300,000

If one searches various Asian newspapers the results are not much different (Table 2).

**Table 2:** Keyword

Keyword	The Star (Malaysia) http://archives.thestar.com.my/search/	China Daily 2000-2012 http://www.chinadaily.com.cn/index.html	The Hindu 1990-2012	Korean Times http://www.koreatimes.co.kr
Nanotechnology	287	267	2076	340
+ Health	82	44	343	52
+ Women	47	6	188	48
+ Disabled people	1	0	3	0
+ People with disabilities	0	0	0	1
+ The poor	8	0	80	0
+ The south	0	0	159	0
+"indigenous people"	0	0	0	0
+ Patient	10	4	102	0

This invisibility also exists for newspapers not based in Asia such as the New York Times (data not shown).

- Part 2: Ability expectations

Science and technology, including nanoscale science and technology, influences and is influenced by various discourses and areas of action. Ableism is one concept and ability expectation is one dynamic that impacts the direction, vision, and application of nanoscale science and technology and vice versa. At the same time policy documents that involved disabled people and that relate to disabled people exhibit ability expectations of disabled people. What ability expectations are exhibited in science and technology direction documents from Asia and in policy documents generated and influenced by disabled people from Asia?

## What is Ableism?

Ableism in its general form is a set of beliefs, processes, and practices that produce, based on one’s abilities, a particular kind of understanding of one’s self, one’s body and one’s relationship with others in terms of one’s species, other species, and one’s environment; additionally, ableism often includes one being judged by others [35,36,38]. Ableism is a favoritism for certain abilities that are projected as essential, while at the same time labeling real or perceived deviation from or lack of these essential abilities as a diminished state of being [35,36]. Those deemed as not having the proper abilities often experience disablism Miller et al. [15], the discriminatory, oppressive, non-accommodating or abusive behavior of people with the ‘required’ abilities toward those who lack certain abilities or exhibit undesirable abilities. Ableism is one of the most societally entrenched and accepted “isms,” and it exists in many forms: biological structure based ableism, cognition based ableism, ableism inherent to a given economic system, and social structure based ableism [35]. Ableism as an analytical framework can be applied to a range of issues including energy security and insecurity [39], human-nature relationship [39], water discourse [40] climate change discourse [37], governance of nanoscale science and technology [38] and issues of inequality and inequity [38].

## Ability Expectations of Disabled People in Asia

What do disabled people want? What are their goals and ability expectations? According to the Durban declaration from the 8th Disabled People’s International (DPI) World Assembly [1], some of the goals and ability expectations are: to be involved in developing strong strategies for the effective implementation of the Convention on the Rights of Persons with Disabilities (CRPD); to be empowered; to participate in political decision making; to be valued and appreciated for their expertise; to participate equally in public life; to increase their capacities; to have access to higher education and training and employment; to be an intrinsic part of the Millennium Development Goals and also of all development programmes post 2015; to be part of development policies and to be taken care of in emergencies and situations of humanitarian risks.

The Bali declaration on the enhancement of the role and participation of persons with disabilities in ASEAN community (ASEAN [2]), which covers Member States of the Association of Southeast Asian Nations (ASEAN), discusses enhancement of well-being and livelihood; providing equitable access to opportunities for human development, social welfare and justice; and reaffirming the potential contribution of persons with disabilities and their important role and participation in the implementation of all action lines under the ASEAN Socio Cultural Community (ASCC). It recognizes the “necessity of persons with disabilities to actively participate in formulating, implementing and evaluating policies related to disability issues in ASEAN region such as the Biwako Millennium Framework and Biwako Plus Five for Action towards an Inclusive, Barrier-free and Rights-based Society in Asia and the Pacific 2003-2012” (ASEAN [2]). The ability to develop “regional statistical indicators in ASEAN to measure the development of vulnerable groups, particularly persons with disabilities is seen as essential” (ASEAN [2]). Ability to have equal opportunities for education, alternative means of communication, to use public facilities and amenities, public transportation, employment, recreation sports is seen as important as being involved in disaster management policies.

## Ability Expectation of Science and Technology Strategies: Two Examples

The Malaysian government [8] in a 2010 Nanotechnology strategy document highlights Nanotechnology as an enabler technology. It is seen to foster sustainable growth and to enhance Malaysia’s environment and standards of living.

Knowledge and resource-sharing, research and development, investment opportunities, commercialisation activities and industrial partnerships are seen as important abilities as are connecting and coordinating the work of scientists, industries and policymakers to enable Nanotechnologies potential for Malaysia. Nanotechnology is seen as a key for improvements in medical care, societal well-being, elimination of man-made pollution and revolutionising agriculture, energy production and utilisation, environmental protection and healthcare as well as aerospace exploration, information technology, national defence and homeland security. The document sees it as essential that Malaysia has ways to generate a competent, skilled and competitive workforce, for which education and training is seen to be critical. The document highlights the importance of the ability to enculturate nanotechnology to become part of the lifestyle and norms in society. The ability expectation of the educational institutions is that it can embed programmes into the curricula packages that encourage continuous student participation in creating solutions for daily problems using Nanoscience. [8].

Sakarindr Bhumiratana, President of the National Science and Technology Development Agency Thailand highlighted in 2007 the National S&T Strategic Plan (2004-2013) [20]. The presentation talks about the ability to generate a middle path between economic growth and a peaceful and harmonious society with the national goal of sustainable growth, green and happiness society [20]. Key Factors to achieve these abilities was public awareness of science and technology. The National Science and Technology Strategic Framework (2004-2013) had the vision of a strong economy with knowledge society and better social well-being talking about sustainable competitiveness, community economy, learning society, quality of life and the environment. It talked about the need for the ability to reduce poverty, to conserve the environment, to effectively utilize biodiversity, to develop renewable energy and improve human Health [20].

## Bringing Disabled People’s and Science and Technology Policy Ability Expectations Together

In principle, ability expectations exhibited by disabled people and the science and technology related documents covered in this article are not in opposition to each other. In short, the documents related to disabled people reflect the desire for ability security whereby “ability security means that one is able to live a decent life with whatever set of abilities one has, and that one will not be forced to have a prescribed set of abilities to live a secure life” [38] and ‘self-identity security’ which “means that one is accepted with one’s set of abilities and that one should not be forced (physically or by circumstance) to accept a perception of oneself one does not agree” [38]. Many if not all of the ability expectations exhibited in the two documents are covered in the UN Convention on the rights of persons with disabilities; to quote one area from the convention,

“Recognizing the valued existing and potential contributions made by persons with disabilities to the overall well-being and diversity of their communities, and that the promotion of the full enjoyment by persons with disabilities of their human rights and fundamental freedoms and of full participation by persons with disabilities will result in their enhanced sense of belonging and in significant advances in the human, social and economic development of society and the eradication of poverty,” [30].

Many of the ability expectations exhibited in the science and technology documents, if achieved, could be instrumental in helping to fulfil the ability expectations of disabled people if they were deployed while also keeping disabled people in mind. However, in order for that to happen, the vision of how science and technology, nanoscale or not, has to move beyond the focus on medical issues and assistive devices linked to the body (as important as this angle is). Many science and technology products can be helpful in changing the physical environment so that it is much more useful for disabled people. However, so far little is available as to this angle of products. The ability expectations espoused in the science and technology documents raise other questions. If new jobs are created, are they accessible to disabled people? Given that they very likely demand certain knowledge, do disabled people have enough access to the education system to gain the knowledge to be competitive in applying for the jobs? So far this has not been the case, as the education statistics available for various countries show. The ability of living in harmony and happiness for sure is important for disabled people, but it comes with an acceptance of who they are and integration into public and societal life which in many places is still rather problematic.

The 2011 World Report on Disability authored by the WHO [41] gives for example the following recommendations:

1. Enable access to all mainstream policies, systems and services
2. Invest in specific programs and services for people with disabilities
3. Adopt a national disability strategy and plan of action
4. Involve people with disabilities
5. Improve human resource capacity
6. Provide adequate funding and improve affordability
7. Increase public awareness and understanding of disability
8. Improve disability data collection
9. Strengthen and support research on disability

However, this is easier asked for than done; for example, it’s easy to put disabled people on science and technology related committees. However, if we put disabled people onto committees without them having the background knowledge on an emerging science and technology field, it’s hard to give credible advice. How is one to advise on whether one envisioned product is more useful than another? How is one to come up as disabled people with a vision of possible products if one does not have the knowledge background as to the possibilities of science and technology fields and which science and technology fields are emerging? The authors submit a first step is to find ways to increase the capacity of disabled people organizations to evaluate the science and technology landscape and what comes down the pipeline; to develop a movement memory of that knowledge so new knowledge can be added constantly to it. The IT field is one area where people with disabilities and their organizations are very knowledgeable and as such can make concrete recommendations. Other emerging science and technology fields such as sensor systems, synthetic biology or other increasingly visible nanoscale sciences and technologies their capacity is still under developed, however. The authors suggest that disabled people in Asian (and other countries, for that matter) develop a catalogue of what abilities they want to have and which science and technology product under what circumstances they think could provide the solution to a given ability expectation. This should cover more than body modifications and ‘medical cures’. What science and technology products could lead to more social cohesion, to the acceptance of disabled people, to their integration in public life? What is needed is a science and technology strategy developed by disabled people and their organizations that is linked to their ability expectations. At the same time, they need to look at science and technology policies in a given country and the products and ability expectations linked to them and ensure that a product developed for even a good purpose like clean water or sanitation is not pushed forward in such a way that its utility is very limited for disabled people. Unfortunately, no guidance from disabled people and their organizations exist in a coherent fashion in most countries. These kinds of documents would have to be posted online on their organizations webpages and constantly be updated; they would have to be ‘living’ documents. The existence of such documents might then lead to more visibility of the voice of disabled people and the topic of utility of science and technology for disabled people in for example the media (beyond a patient angle). Media need material to work with and that material does not exist. The ASEAN Decade of Persons with Disabilities (2011-2020) was just proclaimed and may be this is a good venue to increase S&T literacy of and guidance by disabled people.

What is the role of nanoethicists in regards to for example capacity building of marginalized groups? What is their role in the realizations of science and technology ability expectations in such a way that they are not detrimental to ability expectations of for example disabled people? Do nanoethicists have a role; or more broadly have ethicists covering science and technology issues have a role?

One role that comes to mind is that nanoethicists could be advocates for the ones without voice. Indeed, the role of the ethicist as an advocate is not new. According to the author Self, “the patient advocate model of the medical ethicist in the clinical setting depicts the ethicist as helping to protect patients and defend their rights” [22].

However, some believe that ethicists should not be advocates. According to Spielman the ethicist Veatch voiced the sentiment “that the ethicist playing the role of advocate may lose respect as an ethical analyst (a more widely accepted role among ethicists)” [26].

The Australian now Princeton ethicist Singer stated in 2001:

“But we should reject the view that “the field of bioethics must itself develop a conscience and dedicate itself to advocacy for those who have no money or power to offer this new profession.

Bioethics, as a field or discipline, should not dedicate itself to advocacy for anyone. Its only commitment, as a field, is to pursue knowledge and understanding with integrity and respect for the views of other scholars in the field. It should serve neither those with money and power nor those without it—or rather, it serves all of us best by preserving its independence and freedom of opinion, encouraging open debate and the free exchange of ideas.” [24]

Whether an ethicist should be an advocate and if yes for whom and to what extend is worth of more debate as is the notion evident in the Veatch and Singer quote that ethicists are no advocates. However let’s concede that ethicists should not be advocate for any one group. We submit that ensuring a level playing field of who is heard is not advocacy for a group.

“Community-based participatory research (CBPR) addresses the social justice dimensions of health disparities by engaging marginalized communities, building capacity for action, and encouraging more egalitarian relationships between researchers and communities” [14]. Some see one role of academics as the amplification of the voice of the voiceless [17]. The very field of nanotechnology has as one goal the democratization of the field meaning that stakeholders are heard right at the beginning [28] which in order to be successful has to fulfill certain parameters [25]. As outlined earlier in this paper, disabled people are underrepresented in nanotechnology linked discourses. Indeed, this invisibility and the lack of currency of their views expands to many other science and technology discourses. In general the “public engagement with nanotechnology is less than what we hoped it would be by now” [29].

As to the invisibility of disabled people even if we would ask disabled people for their opinion, how many would have enough knowledge to actually give an opinion that is based on the newest developments in nanotechnology and the discourse around its governance? It seems for the democratization of nanotechnology to work an informed and constantly engaged public is needed that includes disabled people. As to the role of nanoethicists in these democratization efforts; if they agree with the premise, we submit that they have to do their part to further this goal. Indeed, their role is essential as nanoethicists are seen to give advice on the governance of nanoscale science and technology advances. It seems logical that if it is seen as essential that the public gives advice on nanoscale science and technology direction and governance that the public would benefit from a deep knowledge of ethics arguments and ethical reasoning one could apply to the governance. Indeed various ethics education initiatives exist [27] and ethics education is under debate for some time [4,18]. However, we submit an analysis might be needed to ascertain how the content relates to disabled people or other marginalized groups.

Furthermore, if indeed democratization of nanotechnology discourses is a valuable goal it raises the question whether the same democratization is needed for various other academic fields including the ethics field. How diverse is the make-up, the background of ethicists and the angles from which ethics theories are debated and advanced?

Even if we concede that ethicists should not be advocates, do they have an obligation to diversify their field including diversify the background of scholars and the angles from which scholars cover issues so that more diverse voices can be heard and scholars of more diverse background debate with each other?

Susan Sherwin, a Canadian bioethicist, asks “for the expansion of the types of participants engaged in bioethics” [23]. We submit that if participants in the ethics field do not reflect the diversity of populations, a credibility problem exists for the guidance coming out of ethics discourses.

In closing, we submit that to generate a competent public (including disabled people) that is knowledgeable enough to understand the complexity of governance and decision making (especially around science and technology products), as well as to empower the public to make decisions is within the scope of work of ethicists including nanoethicists. We submit further that using ability expectation as a lens of analysis helps to anticipate possible conflicts between different social groups which in turn helps with science and technology governance in general and nanotechnology governance in particular. Finally we submit that having an ethics analysis of ability expectations and the disablism that often follows (field of ableism ethics [38]) as well as an analysis of ability expectations intrinsic to different ethics theories might be a fruitful endeavor.
